# PTEN in triple-negative breast carcinoma: protein expression and genomic alteration in pretreatment and posttreatment specimens

**DOI:** 10.1177/17588359231189422

**Published:** 2023-08-02

**Authors:** Hui Chen, Qingqing Ding, Laila Khazai, Li Zhao, Senthil Damodaran, Jennifer K. Litton, Gaiane M. Rauch, Clinton Yam, Jeffrey T. Chang, Sahil Seth, Bora Lim, Alastair M. Thompson, Elizabeth A. Mittendorf, Beatriz Adrada, Kiran Virani, Jason B. White, Elizabeth Ravenberg, Xingzhi Song, Rosalind Candelaria, Banu Arun, Naoto T. Ueno, Lumarie Santiago, Sadia Saleem, Sausan Abouharb, Rashmi K. Murthy, Nuhad Ibrahim, Mark J. Routbort, Aysegul Sahin, Vicente Valero, William Fraser Symmans, Debu Tripathy, Wei-Lien Wang, Stacy Moulder, Lei Huo

**Affiliations:** Department of Pathology, The University of Texas MD Anderson Cancer Center, Houston, TX, USA; Department of Pathology, The University of Texas MD Anderson Cancer Center, Houston, TX, USA; Department of Pathology, The University of Texas MD Anderson Cancer Center, Houston, TX, USA; Department of Genomic Medicine, The University of Texas MD Anderson Cancer Center, Houston, TX, USA; Department of Breast Medical Oncology, The University of Texas MD Anderson Cancer Center, Houston, TX, USA; Department of Breast Medical Oncology, The University of Texas MD Anderson Cancer Center, Houston, TX, USA; Division of Diagnostic Imaging, The University of Texas MD Anderson Cancer Center, Houston, TX, USA; Department of Breast Medical Oncology, The University of Texas MD Anderson Cancer Center, Houston, TX, USA; Department of Integrative Biology and Pharmacology, The University of Texas Health Science Center at Houston, Houston, TX, USA; Department of Genomic Medicine, The University of Texas MD Anderson Cancer Center, Houston, TX, USA; Department of Oncology, Baylor College of Medicine, Houston, TX, USA; Division of Surgical Oncology, Section of Breast Surgery, Baylor College of Medicine, Houston, TX, USA; Division of Breast Surgery, Department of Surgery, Brigham and Women’s Hospital, Boston, MA, USA; Division of Diagnostic Imaging, The University of Texas MD Anderson Cancer Center, Houston, TX, USA; Division of Pathology and Laboratory Medicine, The University of Texas MD Anderson Cancer Center, Houston, TX, USA; Department of Breast Medical Oncology, The University of Texas MD Anderson Cancer Center, Houston, TX, USA; Department of Breast Medical Oncology, The University of Texas MD Anderson Cancer Center, Houston, TX, USA; Department of Genomic Medicine, The University of Texas MD Anderson Cancer Center, Houston, TX, USA; Division of Diagnostic Imaging, The University of Texas MD Anderson Cancer Center, Houston, TX, USA; Department of Breast Medical Oncology, The University of Texas MD Anderson Cancer Center, Houston, TX, USA; Department of Breast Medical Oncology, The University of Texas MD Anderson Cancer Center, Houston, TX, USA; Division of Diagnostic Imaging, The University of Texas MD Anderson Cancer Center, Houston, TX, USA; Department of Breast Medical Oncology, The University of Texas MD Anderson Cancer Center, Houston, TX, USA; Department of Breast Medical Oncology, The University of Texas MD Anderson Cancer Center, Houston, TX, USA; Department of Breast Medical Oncology, The University of Texas MD Anderson Cancer Center, Houston, TX, USA; Department of Breast Medical Oncology, The University of Texas MD Anderson Cancer Center, Houston, TX, USA; Department of Pathology, The University of Texas MD Anderson Cancer Center, Houston, TX, USA; Department of Breast Medical Oncology, The University of Texas MD Anderson Cancer Center, Houston, TX, USA; Department of Pathology, The University of Texas MD Anderson Cancer Center, Houston, TX, USA; Department of Breast Medical Oncology, The University of Texas MD Anderson Cancer Center, Houston, TX, USA; Department of Pathology, The University of Texas MD Anderson Cancer Center, Houston, TX, USA; Department of Breast Medical Oncology, The University of Texas MD Anderson Cancer Center, Houston, TX, USA; Department of Pathology, The University of Texas MD Anderson Cancer Center, 1515 Holcombe Blvd, Houston, TX 77030, USA

**Keywords:** ARTEMIS, copy number, heterogeneity, immunohistochemistry, neoadjuvant chemotherapy, next-generation sequencing, PTEN, TNBC

## Abstract

**Background:**

Recent advances have been made in targeting the phosphoinositide 3-kinase pathway in breast cancer. Phosphatase and tensin homolog (PTEN) is a key component of that pathway.

**Objective:**

To understand the changes in PTEN expression over the course of the disease in patients with triple-negative breast cancer (TNBC) and whether *PTEN* copy number variation (CNV) by next-generation sequencing (NGS) can serve as an alternative to immunohistochemistry (IHC) to identify PTEN loss.

**Methods:**

We compared PTEN expression by IHC between pretreatment tumors and residual tumors in the breast and lymph nodes after neoadjuvant chemotherapy in 96 patients enrolled in a TNBC clinical trial. A correlative analysis between PTEN protein expression and *PTEN* CNV by NGS was also performed.

**Results:**

With a stringent cutoff for PTEN IHC scoring, PTEN expression was discordant between pretreatment and posttreatment primary tumors in 5% of patients (*n* = 96) and between posttreatment primary tumors and lymph node metastases in 9% (*n* = 33). A less stringent cutoff yielded similar discordance rates. Intratumoral heterogeneity for PTEN loss was observed in 7% of the patients. Among pretreatment tumors, *PTEN* copy numbers by whole exome sequencing (*n* = 72) were significantly higher in the PTEN-positive tumors by IHC compared with the IHC PTEN-loss tumors (*p* < 0.0001). However, PTEN-positive and PTEN-loss tumors by IHC overlapped in copy numbers: 14 of 60 PTEN-positive samples showed decreased copy numbers in the range of those of the PTEN-loss tumors.

**Conclusion:**

Testing various specimens by IHC may generate different PTEN results in a small proportion of patients with TNBC; therefore, the decision of testing one *versus* multiple specimens in a clinical trial should be defined in the patient inclusion criteria. Although a distinct cutoff by which CNV differentiated PTEN-positive tumors from those with PTEN loss was not identified, higher copy number of *PTEN* may confer positive PTEN, whereas lower copy number of *PTEN* would necessitate additional testing by IHC to assess PTEN loss.

**Trial registration:**

NCT02276443.

## Introduction

Recent years have seen great effort to identify effective targeted therapies in addition to the standard, unselected chemotherapy for triple-negative breast cancer (TNBC), an aggressive subtype that comprises approximately 15% of breast cancer. To develop successful targeted therapies, many investigations have focused on the phosphoinositide 3-kinase (PI3K) pathway, whose altered genetic activation is reported in 25–35% of TNBC and contributes to tumor growth, metastasis, and drug resistance.^1–6^ The essential components of the pathway include PI3Ks, Akt serine/threonine kinase family (AKT), mammalian target of rapamycin (mTOR), and phosphatase and tensin homolog (PTEN). PI3Ks are a family of lipid kinases, composed of three classes, among which class I is thought to play a major role in breast cancer. When activated, PI3Ks phosphorylate phosphatidylinositol 4,5-bisphosphate (PtdIns(4,5)P2) to form phosphatidylinositol 3,4,5-trisphosphate (PtdIns(3,4,5)P3). Upon binding to PtdIns(3,4,5)P3, AKT is phosphorylated and activated, which subsequently phosphorylates downstream proteins including mTOR to regulate cell metabolism, survival, proliferation, and migration. One of the key functions of PTEN lies in its inositol polyphosphate 3-phosphatase activity which dephosphorylates PtdIns(3,4,5)P3 to generate PtdIns(4,5)P2; thus, PTEN negatively regulates the PI3K pathway through decreased phosphorylation of AKT.

The most common mechanisms through which the PI3K pathway is activated in TNBC include activating mutations in *PIK3CA* or *AKT1*, and/or inactivating alterations of *PTEN*/loss of PTEN expression.^3,7^ *PIK3CA* encodes the p110 catalytic subunit of class IA PI3Ks, and *AKT1* encodes one of the three isoforms of AKT. *PTEN* was first identified as a tumor suppressor in 1997 in various cancer types including breast cancer.^8,9^ In women with Cowden syndrome, which is linked with *PTEN* germline mutations, there is an estimated 25–30% lifetime risk of breast cancer.^2^ Although *PTEN* mutations have been detected in about 5% of sporadic breast tumors, loss of PTEN expression by immunohistochemistry (IHC) could occur in more than 40%.^2,4,10^ This discrepancy can be explained in part by findings that PTEN protein expression is subject to regulations beyond *PTEN* genomic alterations, through mechanisms such as promoter hypermethylation, microRNA regulation of translation, and posttranslational modifications.^6^ Therefore, evaluating PTEN protein expression is an integral part of assessing PTEN loss in breast cancer. In addition, studies have shown that loss of PTEN expression and *PIK3CA* mutations tend to occur separately in breast tumors including TNBC, even though they are not strictly mutually exclusive.^10,11^ Interestingly, while loss of PTEN expression in breast cancer was highly associated with the triple-negative phenotype in a meta-analysis of over 10,000 patients, PI3K pathway genomic alterations, including *PIK3CA* mutations, occur less in TNBC compared to hormone receptor-positive breast cancer in general.^3,5,12^ These observations further underscore the importance of evaluating PTEN protein expression when assessing the PI3K pathway in TNBC.

There are limited data regarding whether loss of PTEN expression remains consistent in different breast tumor samples over the course of the disease. This knowledge is of clinical importance because in existing clinical trials targeting the PI3K pathway in breast cancer, a fresh biopsy is usually not required to test PTEN expression, and available archival tissue is acceptable for IHC to assess PTEN loss.^11,13–16^ In addition, advances in molecular testing have provided robust information on genomic alterations of cancer biomarkers in many patients diagnosed with breast cancer. We hypothesize that copy number analysis could add value to the evaluation of PTEN status as an alternative to IHC in TNBC. To address the above questions, in the current study, we compared PTEN expression by IHC between pretreatment core biopsies and residual primary breast tumors and lymph node metastases after neoadjuvant chemotherapy in patients enrolled in a single-institution prospective TNBC trial. We also performed a correlative analysis between loss of PTEN expression and *PTEN* genomic alterations measured by next-generation sequencing (NGS) in this cohort.

## Materials and methods

### Human tissue samples

All patients evaluated in this study were enrolled in the ARTEMIS trial (A Robust TNBC Evaluation fraMework to Improve Survival; NCT02276443), which was approved by the Institutional Review Board at MD Anderson Cancer Center. The trial design has been previously described.^17^ Briefly, patients with newly diagnosed stage I–III invasive breast carcinoma that were estrogen receptor negative or low (<10%), progesterone receptor negative or low (<10%), and human epidermal growth factor receptor 2 (HER2) negative^18–20^ were first treated with four cycles of doxorubicin plus cyclophosphamide (AC) chemotherapy, followed by standard paclitaxel-based chemotherapy or an experimental regimen before surgery. Patients in the trial who had surgical resection at MD Anderson by July 2020 were evaluated for the current study. Among 283 patients who had surgery at MD Anderson, 116 had pathologic complete response (pCR) or no invasive breast cancer in the breast (T0) and were therefore excluded from the study since they did not have posttreatment tumor from the breast for evaluation. Among those with residual tumor in the breast, 65 patients were excluded due to lack of material or insufficient tumor for PTEN IHC staining, and six patients were excluded due to staining failure. The remaining 96 patients diagnosed between October 2015 and January 2020, with paired IHC results from pretreatment core biopsies and posttreatment surgical specimens, constituted the study cohort. Lymph node metastases from the posttreatment surgery in 33 patients of this cohort were also stained for PTEN by IHC.

### Immunohistochemistry

The polymeric biotin-free horseradish peroxidase method was used for PTEN IHC staining on a Leica Microsystems Bond III autostainer (Leica Microsystems, Buffalo Grove, IL, USA). In each case, one whole slide unstained tissue section of 4 μm thickness that had been prepared from a representative paraffin block of the invasive breast carcinoma was subjected to heat-induced epitope retrieval with Tris-EDTA buffer for 20 min at 100°C. Slides were then incubated with mouse monoclonal antibody to PTEN (clone 6H2.1, 1:100, Dako, Carpinteria, CA, USA). The Refine Polymer Detection kit was used to detect bound antibody, with 3,3-diaminobenzidine serving as the chromogen (Leica Microsystems). Slides were counterstained with Mayer’s hematoxylin. Results were evaluated with appropriate positive and negative tissue controls.

Two scoring approaches were applied. Scoring system 1: Nuclear or cytoplasmic staining of any intensity in any proportion of invasive tumor cells was considered positive, and no staining in the invasive tumor cells was considered loss. Staining was recorded as equivocal if it was difficult to distinguish from nonspecific background staining. In posttreatment samples, staining was recorded as heterogeneous as a subgroup of positive staining when there was loss of PTEN in well-demarcated areas/clusters of tumor cells, regardless of the extent of the loss; however, this did not include the presence of scattered negatively stained individual cells. Scoring system 2: Nuclear or cytoplasmic staining of any intensity in >50% of invasive tumor cells was considered high, and no staining in ⩾50% of invasive tumor cells was considered low. Equivocal staining was defined as described in Scoring system 1. On the basis of these approaches, PTEN expression was evaluated by breast pathologists (HC, QD, LH, and LK). Consensus was reached at multiheaded microscopes for cases in question.

### NGS – whole exome sequencing

Whole exome sequencing (WES) was performed with pretreatment samples from 76 patients and posttreatment samples from 17 patients. Genomic DNA was extracted from fresh tumor tissue; matched blood samples were used as germline controls. Exome capture was performed on 500 ng of DNA per sample based on the Kapa Hyper Prep kit using the Agilent Human All Exon bait kit according to the manufacturer’s instructions (KAPA Biosystems, Wilmington, MA, USA). WES was performed on the Illumina HiSeq 2500 sequencing platform. Paired-end sequencing reads in FASTQ format were generated from BCL raw data using Illumina CASAVA software (v1.8.2) (Illunima, San Diego, CA, USA). The reads were aligned to the hg19 human reference genome using BWA (v0.7.5).^21^ The duplicate reads were removed using Picard (http://broadinstitute.github.io/picard/), and local realignments were performed using GATK (v4.1.1.0).^22^ The binary alignment map (BAM) files were then used for downstream analysis.

#### Mutation calling and copy number variation identification

MuTect (v1.1.4)^23^ was used to identify somatic point variants, and Pindel (v0.2.4)^24^ was used to identify somatic insertions and deletions. A series of post-calling filters were applied for somatic mutations including the following: (a) total read count in a tumor sample of ⩾20, (b) total read count in a germline sample of ⩾10, (c) a variant allele frequency of ⩾0.02 in tumor sample and ⩽0.02 in matched normal sample, and (d) a population frequency threshold of 1% to filter out common variants in the databases of dbSNP129,^25^ 1000 Genomes Project,^26^ Exome Aggregation Consortium,^27^ and ESP6500.^28^ To understand the functional impact of detected variants, we annotated them using Annovar^29^ and dbNSFP^30^ and compared them with dbSNP,^31^ ClinVar,^32^ Catalogue of Somatic Mutations in Cancer (COSMIC),^33^ and The Cancer Genome Atlas (TCGA) databases.

Copy number variation (CNVs) were identified using an in-house algorithm called ExomeCN. The copy number log2 ratios of tumor *versus* matched normal tissue were calculated across the entire capture regions and then subjected to segmentation using the Circular Binary Segmentation method.^34^

#### Parameters for WES and IHC comparison

We explored different parameters for the comparison between WES and IHC results. In all, 10 probes were designed to cover the *PTEN* exon regions in WES, and the genomic locations of the last two probes overlapped with the PTEN antibody binding region in IHC. Therefore, for WES-IHC comparison in pretreatment specimens, we tried three parameters for the log2 ratio obtained from WES: the average log2 ratio of all 10 probes, the average log2 ratio of the last two probes, and the log2 ratio of the last probe on *PTEN*. To exclude the possibility of chromosome-level loss or gain, the average log2 ratios of chromosomes 8–12 were also calculated to estimate the baseline level of chromosome 10. We first excluded two samples with *PTEN* mutations, and then compared the log2 ratios between the samples with positive PTEN by IHC and those with PTEN loss by IHC. A cutoff value of log2 ratio −0.4 was applied based on previous studies with similar analytical approaches.^35^ The average log2 ratio of all probes on *PTEN* was determined to provide the best differentiation between the two IHC PTEN expression groups.

### Statistical analysis

The correlation between IHC results and CNV was determined using a two-sample Wilcoxon test. The *p* values of 0.05 or less were considered statistically significant.

## Results

### PTEN expression by IHC in pretreatment and posttreatment tumors (Scoring system 1)

#### Primary tumors

**Figure 1.**
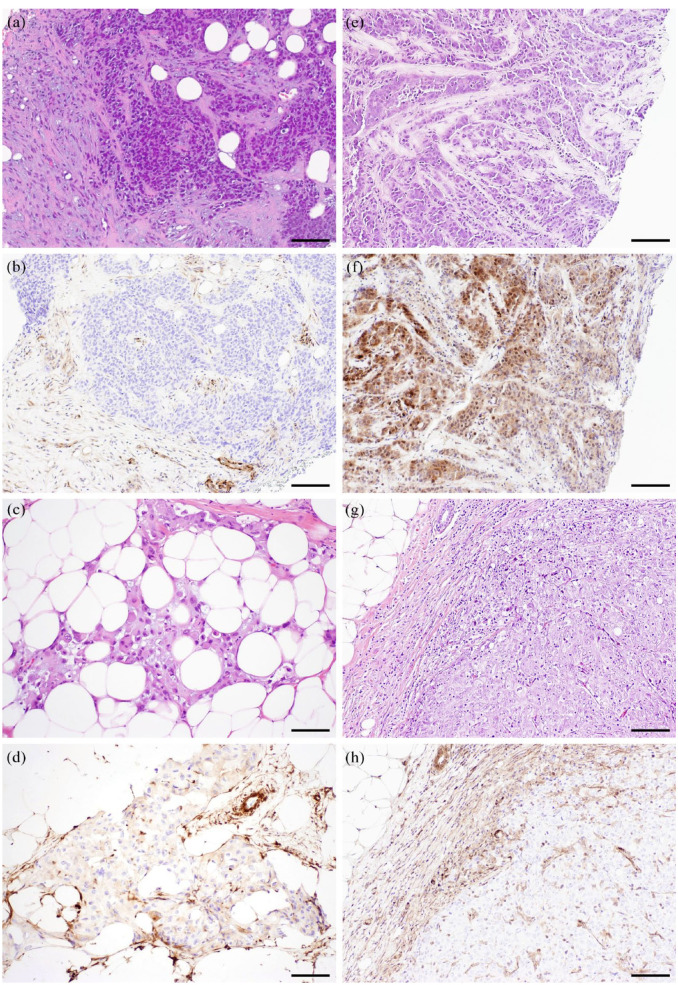
Photomicrographs of representative cases with changes in PTEN immunohistochemical staining between pretreatment and posttreatment primary tumors. (a)–(d) Paired specimens from one patient: (a) pretreatment tumor, hematoxylin and eosin stain; (b) pretreatment tumor with loss of PTEN staining; (c) posttreatment tumor, hematoxylin and eosin stain; (d) posttreatment tumor, positive PTEN stain. (e)–(h) Paired specimens from another patient: (e) pretreatment tumor, hematoxylin and eosin stain; (f) pretreatment tumor, positive PTEN stain; (g) posttreatment tumor, hematoxylin and eosin stain; (h) posttreatment tumor with loss of PTEN staining. Scale bar, 100 µm. PTEN, phosphatase and tensin homolog.

The final analysis included 96 patients with paired pretreatment core biopsies and posttreatment surgical specimens. The clinicopathologic characteristics of the patients are described in Supplemental Table 1. Using Scoring system 1, concordance between pretreatment and posttreatment primary tumors was reached in 89 patients (93%), including 75 (78%) with positive PTEN and 14 (15%) with PTEN loss (Table 1). In five patients, there was a positive-to-loss or loss-to-positive change between the pretreatment and posttreatment specimens (Figure 1).

**Table 1.** Comparison of PTEN staining in paired pretreatment and posttreatment primary tumors.

PTEN staining (*n* = 96)		Pretreatment			
		Positive	Loss	Equivocal	Total
Posttreatment	Positive	75	4	2	81 (84%)
	Loss	1	14	0	15 (16%)
	Total	76 (79%)	18 (19%)	2 (2%)	96

Posttreatment tumors with heterogeneous staining were considered positive.

PTEN, phosphatase and tensin homolog.

Heterogeneous staining was evaluated only in posttreatment tumors because sampling was more representative compared with pretreatment core biopsies. Seven posttreatment tumors (7%) showed heterogeneous staining. Among them, three tumors showed predominantly negative staining, with focal weak positive staining [Supplemental Figure 1(a)–(j)]. One tumor showed loss of staining in approximately 50% of the tumor area [Supplemental Figure 1(k)–(n)]. The other three tumors showed focal loss. None of the cases with heterogeneous staining showed a significant morphologic difference between the negatively and positively stained components. Two of the tumors showed PTEN loss in the corresponding pretreatment tumors (Table 2).

**Table 2.** Posttreatment primary tumors with heterogeneous PTEN staining.

Case #	Extent of PTEN loss in posttreatment tumor (%)	PTEN status in pretreatment core biopsy
1	>70	Loss
2	>70	Positive
3	>70	Positive
4	50	Loss
5	15	Positive
6	<5	Positive
7	<5	Positive

PTEN, phosphatase and tensin homolog.

#### Posttreatment primary tumors and lymph node metastases

PTEN staining was performed on the posttreatment residual lymph node metastases in 33 patients. The results in comparison to the corresponding posttreatment primary tumors are summarized in Table 3. Concordance between residual primary tumor and lymph node metastasis was seen in 30 (91%) patients. In the remaining three patients, there was a change from positive to loss or loss to positive (Supplemental Figure 2). In each of these three patients, the pretreatment primary tumor had concordant staining with the posttreatment primary tumor. Lymph node metastases were available for PTEN IHC in two of the patients whose primary tumors showed heterogeneous staining. In both patients, the lymph node metastases showed positive staining.

**Table 3.** Comparison of PTEN staining in paired posttreatment primary tumor and lymph node metastasis.

PTEN staining (*n* = 33)		Lymph node metastasis		
		Positive	Loss	Total
*Primary tumor*	Positive	25	2	27 (82%)
	Loss	1	5	6 (18%)
	Total	26 (79%)	7 (21%)	33

PTEN, phosphatase and tensin homolog.

**Figure 2.**
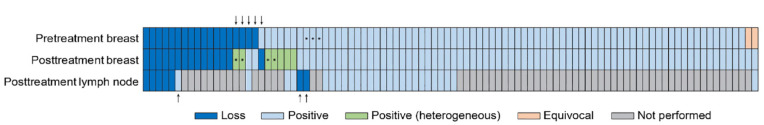
Summary of PTEN IHC results using no staining as cutoff for loss (Scoring system 1). Each vertical box represents one tumor specimen. Arrows above the diagram indicate the cases where PTEN was discordant between pretreatment and posttreatment primary tumors. Arrows below the diagram indicate the cases where PTEN was discordant between posttreatment primary tumors and lymph node metastases. Asterisks mark the tumors that changed PTEN category with Scoring system 2. IHC, immunohistochemistry; PTEN, phosphatase and tensin homolog.

The PTEN staining results in the pretreatment and posttreatment specimens are summarized schematically in Figure 2.

### PTEN expression by IHC in pretreatment and posttreatment tumors (Scoring system 2)

Given the lack of standard scoring criteria for PTEN loss, we applied a second, less stringent scoring approach to explore the impact of scoring on PTEN status. In contrast to the first scoring approach, in the second one, if 50% or more invasive tumor cells stained negative for PTEN, the result was considered low PTEN expression. As a result, three cases among the pretreatment tumors (3/96, 3%), and four cases among the posttreatment primary tumors (4/96, 4%) changed from positive in Scoring system 1 to low in Scoring system 2. The four cases whose posttreatment tumor status changed corresponded to the first four cases with heterogeneous staining in Table 2. There was no change in category among the lymph node metastases. Overall, this second scoring approach yielded a concordance of 90% (86/96) between pretreatment and posttreatment primary tumors, and the same concordance (91%) between posttreatment primary tumors and lymph node metastases as found when using Score system 1. The staining results in the pretreatment and posttreatment specimens using Score system 2 are summarized schematically in Supplemental Figure 3.

### PTEN copy number variation by NGS

WES was carried out on fresh tissue of pretreatment primary tumors in 76 patients, and on fresh posttreatment primary tumor tissue in 17 patients. IHC results from Scoring system 1 were used for the correlative analysis below.

#### Correlation between PTEN expression by IHC and CNV in pretreatment tumors

**Figure 3.**
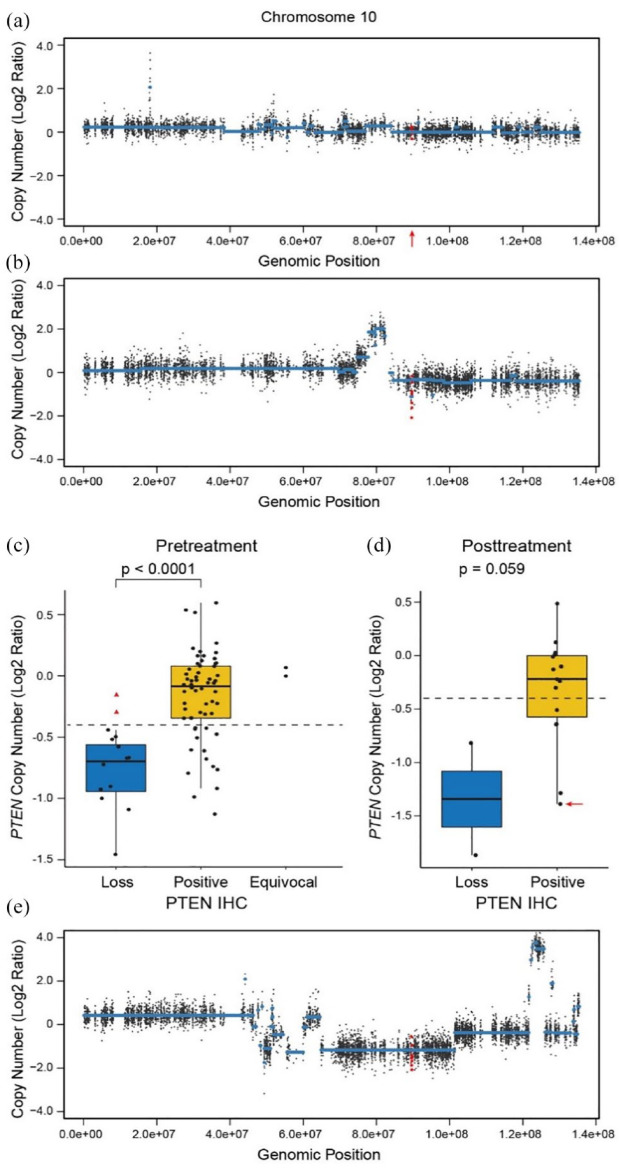
Copy number analysis by WES. (a) and (b) Graphic display of copy number analysis of *PTEN* in representative pretreatment tumors. (a) A tumor with normal *PTEN* copy number detected, with the location of the *PTEN* gene marked by a red arrow. (b) A tumor with decreased *PTEN* copy number. (c) and (d) Box plots of copy number analysis of *PTEN* in correlation with PTEN IHC. (c) Pretreatment primary tumors. The red triangles indicate the two tumors with a four-nucleotide deletion and a single-nucleotide nonsense mutation (not included in the statistical analysis of the positive and loss groups). (d) Posttreatment primary tumors. The red arrow indicates the tumor with heterogeneous IHC staining. In (c) and (d), the dashed line indicates the position of −0.4. (e) Graphic display of copy number analysis of *PTEN* in the posttreatment tumor with heterogeneous PTEN IHC staining marked in (d). The *PTEN* probes are indicated in red in (a), (b), and (e). IHC, immunohistochemistry; PTEN, phosphatase and tensin homolog; WES, whole exome sequencing.

Somatic mutations were identified in two pretreatment tumors by WES. One tumor had a four-nucleotide deletion resulting in an immediate stop codon (c.955_958del p.T319*), and the other had a single-nucleotide nonsense mutation (c.C195G p.Y65*). Both tumors showed PTEN loss by IHC. Correlative CNV analysis was performed on the 72 tumors after excluding the two tumors with mutations and the two tumors with equivocal PTEN by IHC. The average log2 ratio of all probes on *PTEN* provided the best differentiation between the tumors with positive IHC (*n* = 60) and those with PTEN loss (*n* = 12) [Figure 3(a) and (b)]. The IHC-positive group had significantly higher *PTEN* copy numbers compared with the IHC PTEN-loss group (*p* < 0.0001). The average log2 ratio by WES was −0.17 (equivalent of copy number 1.78) in the IHC-positive tumors, and −0.79 (equivalent of copy number 1.16) in those with IHC PTEN loss. However, while all the IHC PTEN-loss tumors had a log2 ratio of below −0.4 by WES, 14 IHC-positive tumors also had a log2 ratio below −0.4 by WES [Figure 3(c)]. Thus, using WES, we could not entirely distinguish PTEN-positive and PTEN-loss tumors based on CNV. Notably, all the tumors with a log2 ratio of above −0.4 by WES were PTEN positive by IHC. They may represent a group for which positive PTEN status can be determined by WES without additional IHC testing.

#### Correlation between PTEN expression by IHC and CNV in posttreatment tumors

With the parameters derived from the CNV analysis of the pretreatment tumor, we further examined *PTEN* copy number in 17 patients who had WES performed on their posttreatment primary tumors. Among them, two had PTEN loss, and the other 15 showed positive PTEN by IHC, including the one with heterogeneous staining with 50% PTEN loss. While there was a trend of lower copy numbers in the PTEN-loss group (*p* = 0.059), the copy numbers of the two groups overlapped, similar to those of the pretreatment tumors [Figure 3(d)]. Interestingly, the tumor with heterogeneous IHC staining showed a relatively low copy number, with a log2 ratio (−1.39) near the average of the tumors with PTEN loss (−1.34) [Figure 3(d) and (e)].

## Discussion

In this study, we have shown a less than 10% discordance rate for PTEN expression detected by IHC between samples before and after neoadjuvant therapy, and between the primary tumors and lymph node metastases after neoadjuvant therapy in patients with early-stage TNBC enrolled in the ARTEMIS trial. By NGS, the IHC PTEN-positive group had significantly higher *PTEN* copy numbers compared with the IHC PTEN-loss group. PTEN protein expression and genomic alteration is an integral part of the PI3K pathway evaluation. Several clinical trials have explored the use of PI3K or AKT inhibitors in TNBC.^7,11,13–16,36,37^ In BELLE-4, a randomized adaptive phase II/III trial, the pan-class I PI3K inhibitor buparlisib was tested in combination with paclitaxel in HER2-negative, locally advanced or metastatic breast cancer, which did not improve progression-free survival (PFS) in the full or PI3K pathway-activated study population, including 50 and 17 patients with TNBC, respectively.^13^ Ipatasertib, a selective small-molecule inhibitor of all three isoforms of AKT, in combination with paclitaxel, was not found to be associated with a statistically significant increase of pCR in the randomized phase II FAIRLANE trial in the neoadjuvant setting for early TNBC, including *PIK3CA/AKT/PTEN*-altered and PTEN-low subgroups.^14^ In contrast, in the randomized phase II LOTUS trial, ipatasertib plus paclitaxel as first-line therapy for metastatic TNBC led to longer PFS and overall survival compared with placebo plus paclitaxel. The difference in PFS was significant in patients with *PIK3CA/AKT/PTEN*-altered tumors, but not significant in those with *PIK3CA/AKT/PTEN*-non-altered tumors.^15,16^ Similarly, capivasertib, a selective small-molecule inhibitor against the three AKT isoforms, combined with paclitaxel as first-line therapy for metastatic TNBC, resulted in longer PFS and overall survival compared with placebo plus paclitaxel in the PARK trial, with the benefit more pronounced in patients with *PIK3CA/AKT/PTEN*-altered tumors.^7^ However, the results from cohort A of the phase III IPATunity130 trial, presented in an abstract form, failed to support an improved PFS demonstrated in the phase II LOTUS trial.^36^ In addition, monotherapy of another selective pan-AKT inhibitor, MK-2206, demonstrated limited clinical activity in a phase II trial of metastatic/advanced breast cancer patients with *PIK3CA, AKT*, or *PTEN* mutations, or PTEN loss in their tumors, including nine TNBCs.^11^ Nevertheless, in the I-SPY 2 trial of high-risk early-stage breast cancer patients, MK-2206 combined with standard neoadjuvant chemotherapy showed a 96.7% probability of superiority to chemotherapy alone in TNBC.^37^ Taken together, the data from these trials suggest that targeting the PI3K pathway is a promising approach in TNBC and needs further investigation.

By WES, Luo *et al.* demonstrated intratumoral heterogeneity in *PTEN* somatic mutations in inflammatory breast cancer.^38^ By IHC, intratumoral heterogeneity in PTEN expression has been shown by some authors but not others,^10,39^ although the use of tissue microarrays in these studies may compromise the accuracy of the results to some extent.^40^ In our study using whole slide sections from the post-neoadjuvant therapy surgical specimens, with heterogeneity defined as loss of PTEN in well-demarcated areas/clusters of tumor cells, we identified 7 out of 96 tumors with two distinct cell populations of PTEN expression. Understanding the frequency of intratumoral heterogeneity is important because it could cause altered expression detected in different samples throughout the course of the disease in a patient, owing to either clonal selection or limited tumor sampling. In our study, we observed changes between pretreatment and posttreatment primary tumors in five patients and between posttreatment primary tumors and lymph node metastases in an additional three patients (totaling 8/96, or 8%). Similarly, PTEN changes were reported in the MK-2206 monotherapy trial in metastatic/advanced breast cancer patients, where PTEN expression by IHC was assessed in both archival samples and samples procured just prior to MK-2206 treatment in 22 patients.^11^ In all, 19 (86%) had concordant results, 2 had PTEN loss in the archival samples and no PTEN loss in the recent samples, and 1 had PTEN loss in the recent sample and no PTEN loss in the archival sample. In addition, 10 patients had paired pretreatment and on-treatment biopsies in that study. Among them, three (30%) had discordant PTEN results by IHC: two had PTEN loss with treatment while the baseline pretreatment sample had no loss, and one patient had PTEN loss pretreatment but upregulation of PTEN expression with treatment. Thus, our findings of heterogeneous PTEN expression within individual tumors provide the basis for multiple testing if clinically indicated.

Among 25 IHC studies included in a meta-analysis of loss of PTEN expression in breast cancer, 10 studies considered any staining PTEN positive, eight studies had a range between 5% and 15% regardless of intensity as the cutoff for PTEN positivity, five used H-scores of 30–100 as the cutoff to include both intensity and extent of staining in the evaluation, and two required moderate or strong staining to any extent to be counted as positive.^12^ The diversity in criteria for PTEN loss was also reflected in the clinical trials targeting the PI3K pathway. In the BELLE-4 trial, 1+ staining in ⩽10% of tumor cells was set for PTEN loss. In the FAIRLANE and the LOTUS trials, PTEN low was defined as no staining in ⩾50% of tumor cells. In the MK-2206 monotherapy trial, faint staining in up to 50% of tumor cells was allowed to designate a tumor as having PTEN loss. None of these trials demonstrated a significant difference between the PTEN-loss/low and the PTEN-positive populations in response to the tested drug.^11,13,14^ We applied two scoring approaches in the current study: one used the most stringent criterion of no staining in invasive tumor cells to define PTEN loss, and the other adopted the most liberal cutoff of no staining in ⩾50% of tumor cells to define PTEN low, as in the FAIRLANE and LOTUS trials. Even though the cutoffs in the two scoring approaches seemed quite different, to our surprise, the impact was rather small: only 3% of pretreatment and 4% of posttreatment primary tumors had a change in PTEN category due to the different cutoffs, leading to a 3% increase in the discordant rate between pretreatment and posttreatment primary tumors. There was no change in PTEN category in the lymph node metastases due to the change in cutoff. Thus, the various scoring methods defining PTEN loss in the above clinical trials likely would not have made a major difference in the outcomes.

Our study had several limitations. First, to compare PTEN expression between paired pretreatment and posttreatment tumors, only patients with sufficient residual tumor material for IHC after neoadjuvant therapy were included in the study, which was a selection bias. However, because additional treatment is more likely to be given to the patients with significant residual disease after neoadjuvant therapy, our cohort could represent a clinically relevant subset. Second, the pretreatment core biopsy material contained a small proportion of the tumor, which could introduce potential bias due to limited sampling. The comparison between pretreatment core biopsy and posttreatment surgical material was complicated by sampling as well. Furthermore, while mutation analysis has been well established in the clinical Laboratory Improvement Amendments setting, CNV by NGS assay is still under exploration. In the LOTUS trial including patients with metastatic TNBC, among 15 patients who had a *PTEN*-inactivating alteration and a PTEN IHC test, 14 (93%) showed low PTEN expression. In contrast, only 14 of 37 (38%) patients with low PTEN expression by IHC showed a *PTEN*-inactivating alteration by NGS.^15^ In our study, PTEN loss by IHC was significantly associated with decreased PTEN copy number by WES. However, a considerable overlap was noted, which could be attributed to a combination of intratumoral heterogeneity, sampling variation between IHC and NGS, a dilution effect from different amounts of normal tissue included in each sample, and bioinformatic algorithms. To improve the accuracy of the genomic assessment of copy number, we may explore strategies involving deeper DNA profiling or integration with other types of data in future studies. Additional bioinformatic algorithms with refined copy number analysis may also be investigated in a larger dataset.

In summary, our study demonstrated that in this group of patients with early-stage TNBC enrolled in a single-institution prospective trial and treated with neoadjuvant therapy, PTEN expression was discordant between pretreatment and posttreatment primary tumor samples in 5% (*n* = 96) of patients and between posttreatment primary tumor and lymph node metastasis in 9% (*n* = 33) using a stringent cutoff for PTEN IHC scoring. A less stringent cutoff yielded similar discordance rates (8% and 9%, respectively). Intratumoral heterogeneity for PTEN loss was observed in 7% of the patients. PTEN expression by IHC was significantly associated with CNV by WES. Although we could not find a cutoff by which CNV separated PTEN-positive tumors from those with PTEN loss, our data suggest there could be a cutoff for CNV where patients with higher PTEN copy numbers can be spared of additional IHC testing because they are unlikely to have PTEN loss, and those with lower PTEN copy numbers would need PTEN IHC testing to confirm PTEN loss. Additional studies and improved platforms of CNV analysis may help validate our results. With the understanding that testing various specimens may generate different PTEN results in a small proportion of patients, the selection of specimens for testing as well as how to use results generated from different specimens from a patient is determined by whether the goal is to include patients with PTEN loss in any specimen or to treat the current tumor based on its PTEN status in clinical trials. It would be interesting to investigate the significance of intratumoral heterogeneity for PTEN loss in clinical trials targeting the PI3K pathway. Recently, PTEN has been shown to play an important role in the composition and function of the tumor microenvironment; therefore, loss of PTEN may impact response to immunotherapy.^41^ Large clinical trials are needed to determine the most relevant PTEN assessment based on treatment response.

## Supplemental Material

sj-docx-1-tam-10.1177_17588359231189422 – Supplemental material for PTEN in triple-negative breast carcinoma: protein expression and genomic alteration in pretreatment and posttreatment specimens Click here for additional data file. Supplemental material, sj-docx-1-tam-10.1177_17588359231189422 for PTEN in triple-negative breast carcinoma: protein expression and genomic alteration in pretreatment and posttreatment specimens by Hui Chen, Qingqing Ding, Laila Khazai, Li Zhao, Senthil Damodaran, Jennifer K. Litton, Gaiane M. Rauch, Clinton Yam, Jeffrey T. Chang, Sahil Seth, Bora Lim, Alastair M. Thompson, Elizabeth A. Mittendorf, Beatriz Adrada, Kiran Virani, Jason B. White, Elizabeth Ravenberg, Xingzhi Song, Rosalind Candelaria, Banu Arun, Naoto T. Ueno, Lumarie Santiago, Sadia Saleem, Sausan Abouharb, Rashmi K. Murthy, Nuhad Ibrahim, Mark J. Routbort, Aysegul Sahin, Vicente Valero, William Fraser Symmans, Debu Tripathy, Wei-Lien Wang, Stacy Moulder and Lei Huo in Therapeutic Advances in Medical Oncology

## Declarations

**Ethics approval and consent to participate:** Written informed consents were obtained from enrolled patients. The study was conducted under protocol 2014-0185 approved by the Institutional Review Board of MD Anderson Cancer Center.

**Consent for publication:** Written informed consents including consents for publication were obtained from enrolled patients.

**Availability of data and materials:** The data used for this study will be made available upon reasonable request from the corresponding author.
