# Supplementary material for: PTEN in triple-negative breast carcinoma: protein expression and genomic alteration in pretreatment and posttreatment specimens
Source: Ther Adv Med Oncol. 2023 Aug 2;15:17588359231189422. doi: 10.1177/17588359231189422 (PMC10399250; doi:10.1177/17588359231189422)
Supplement: sj-docx-1-tam-10.1177_17588359231189422 – Supplemental material for PTEN in triple-negative breast carcinoma: protein expression and genomic alteration in pretreatment and posttreatment specimens [file sj-docx-1-tam-10.1177_17588359231189422.docx]

**Supplementary Table 1.** Patient clinicopathologic characteristics

|  | **No. (%)** |
| --- | --- |
| **Histologic type** |  |
| Ductal | 75 (78) |
| Lobular | 1 (1) |
| Metaplastic | 20 (21) |
| **Histologic grade** |  |
| 1-2 | 16 (17) |
| 3 | 80 (83) |
| **T stage** |  |
| T1 | 10 (10) |
| T2 | 57 (59) |
| T3 | 19 (20) |
| T4 | 10 (10) |
| **N stage** |  |
| N0 | 56 (58) |
| N1 | 16 (17) |
| N2 | 3 (3) |
| N3 | 21 (22) |
| **Clinical TNM stage** |  |
| I | 8 (8) |
| II | 56 (58) |
| III | 32 (33) |
| **Residual cancer burden status** |  |
| I | 7 (7) |
| II | 66 (69) |
| III | 23 (24) |

All patients’ characteristics were from pretreatment, except for residual cancer burden status.


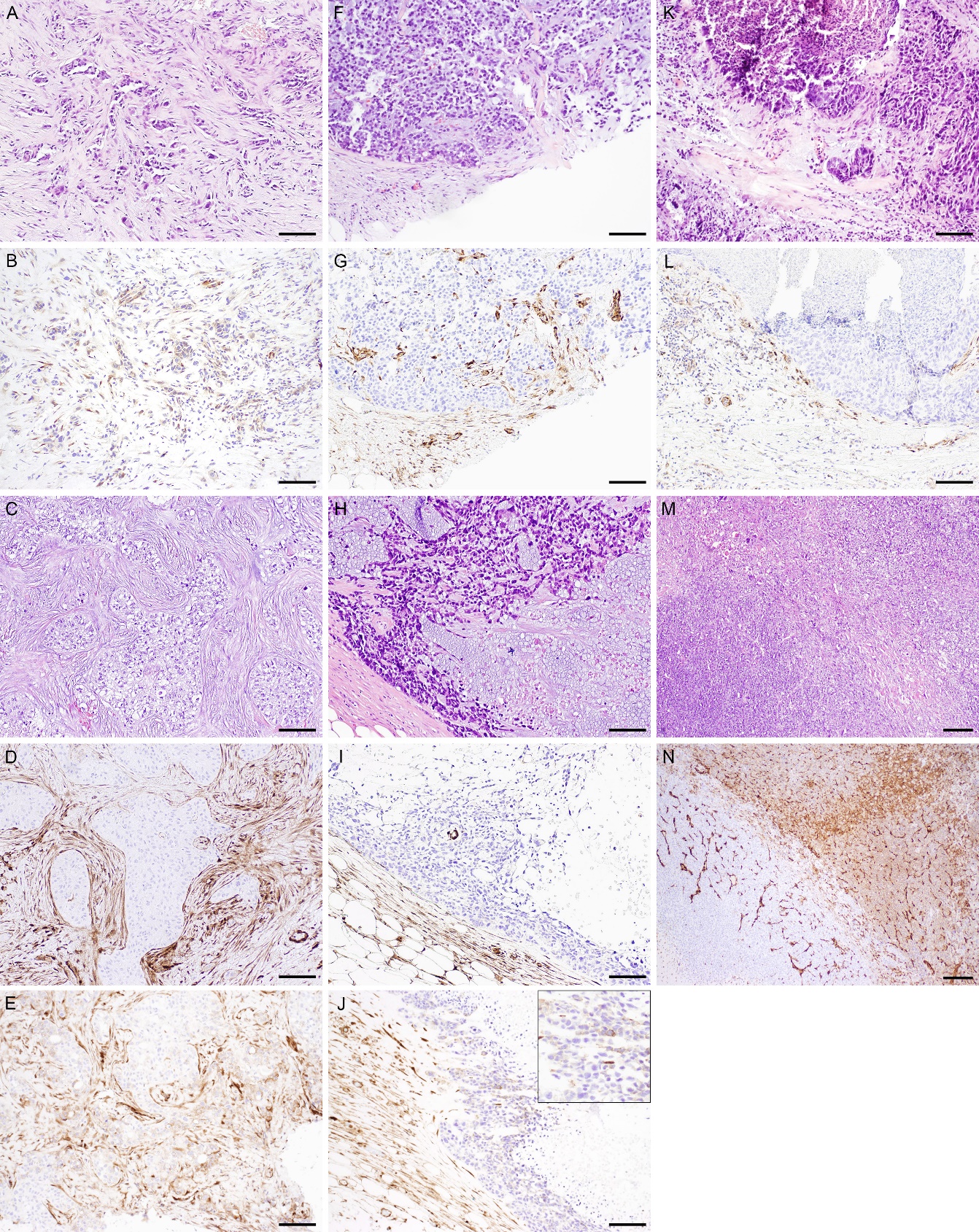


**Supplementary Figure S1**. Photomicrographs of representative cases with heterogeneous PTEN staining. A-E, paired specimens from one patient with predominantly negative staining in the posttreatment primary tumor. A, pretreatment tumor, hematoxylin and eosin stain; B, pretreatment tumor, positive PTEN stain; C, posttreatment tumor, hematoxylin and eosin stain; D, posttreatment tumor, an area with PTEN loss; E, focal weak positive PTEN stain in the same posttreatment tumor. F-J, paired specimens from another patient with predominantly negative staining in the posttreatment primary tumor. F, pretreatment tumor, hematoxylin and eosin stain; G, pretreatment tumor with loss of PTEN staining; H, posttreatment tumor, hematoxylin and eosin stain; I, posttreatment tumor, an area with loss of PTEN staining; J, focal weak positive PTEN stain in the same posttreatment tumor. Inset in J, higher magnification of the focal positive area. K-N, paired specimens from one patient with 50% negative staining in the posttreatment primary tumor. K, pretreatment tumor, hematoxylin and eosin stain; L, pretreatment tumor with loss of PTEN staining; M, posttreatment primary tumor, hematoxylin and eosin stain; N, posttreatment primary tumor with well demarcated areas of positive PTEN stain (upper right) and PTEN loss (lower left); Scale bar, A-L, 100 µm; M and N, 200 µm.


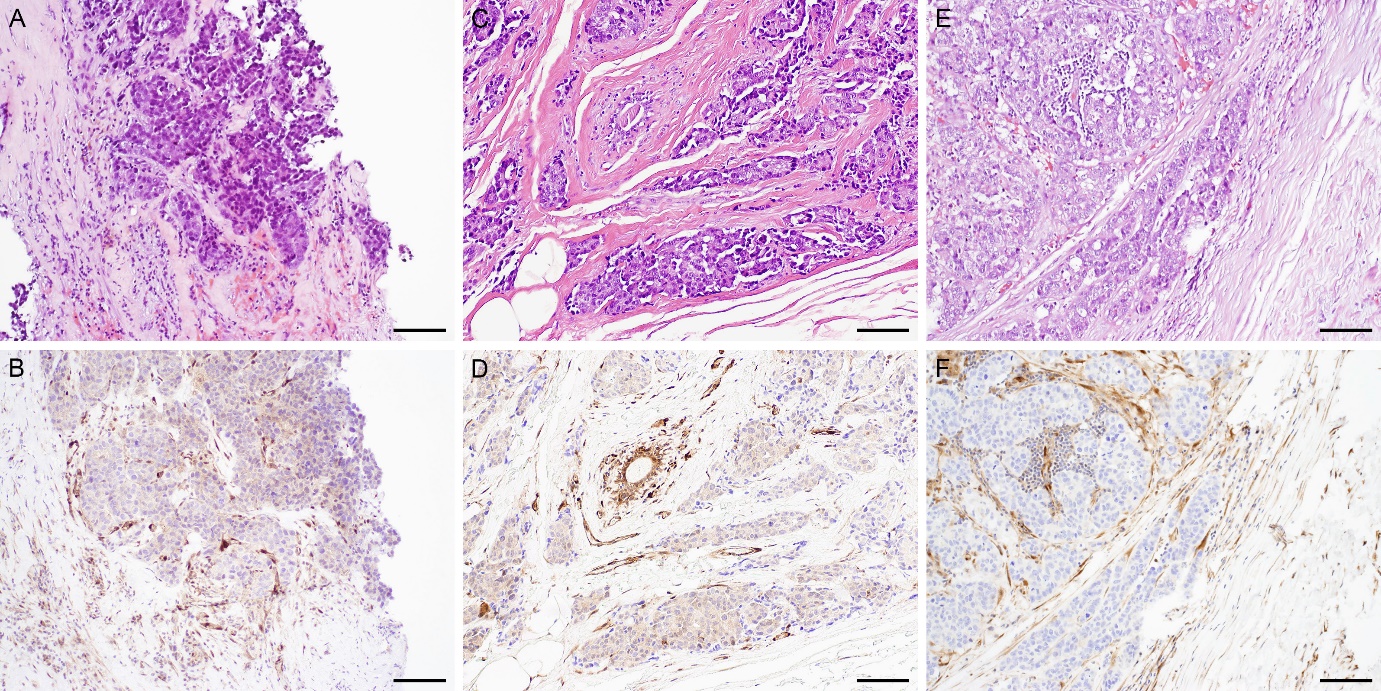


**Supplementary Figure S2**. Photomicrographs of a representative case with different PTEN staining between the posttreatment primary tumor and lymph node metastasis. A, pretreatment primary tumor, hematoxylin and eosin stain; B, pretreatment primary tumor, positive PTEN stain; C, posttreatment primary tumor, hematoxylin and eosin stain; D, posttreatment primary tumor, positive PTEN stain; E, posttreatment lymph node metastasis, hematoxylin and eosin stain; F, posttreatment lymph node metastasis with loss of PTEN staining. Scale bar, 100 µm.

**Supplementary Figure S3.** Summary of PTEN immunohistochemistry results using 50% staining as cutoff for low PTEN (Scor-ing system 2). Each vertical box represents one tumor specimen. Arrows above the diagram indicate the PTEN discordant cases between pretreatment and posttreatment primary tumors. Arrows below the diagram indicate the PTEN discordant cases between posttreatment primary tumors and lymph node metastases. Asterisks mark the tumors that changed PTEN category from Scoring system 1.
